# Interleukin-1 and Tumor Necrosis Factor-α Trigger Restriction of Hepatitis B Virus Infection via a Cytidine Deaminase Activation-induced Cytidine Deaminase (AID)*

**DOI:** 10.1074/jbc.M113.501122

**Published:** 2013-09-11

**Authors:** Koichi Watashi, Guoxin Liang, Masashi Iwamoto, Hiroyuki Marusawa, Nanako Uchida, Takuji Daito, Kouichi Kitamura, Masamichi Muramatsu, Hirofumi Ohashi, Tomoko Kiyohara, Ryosuke Suzuki, Jisu Li, Shuping Tong, Yasuhito Tanaka, Kazumoto Murata, Hideki Aizaki, Takaji Wakita

**Affiliations:** From the ‡ Department of Virology II, National Institute of Infectious Diseases, Tokyo 162-8640, Japan,; the § Department of Molecular Genetics, Kanazawa University Graduate School of Medical Science, Kanazawa 920-8640, Japan,; the ¶ Department of Gastroenterology and Hepatology, Kyoto University Graduate School of Medicine, Kyoto 606-8507, Japan,; the ‖ Liver Research Center Rhode Island Hospital, Warren Alpert School of Medicine, Brown University, Providence, Rhode Island 02903,; the ** Department of Virology and Liver Unit, Nagoya City University Graduate School of Medicinal Sciences, Nagoya 467-8601, Japan, and; the ‡‡ Research Center for Hepatitis and Immunology, National Center for Global Health and Medicine, Ichikawa 272-8516, Japan

**Keywords:** Innate Immunity, Interferon, Interleukin, Tumor Necrosis Factor (TNF), Virus, AID, APOBEC3G, HBV, HepaRG, Deaminase

## Abstract

Virus infection is restricted by intracellular immune responses in host cells, and this is typically modulated by stimulation of cytokines. The cytokines and host factors that determine the host cell restriction against hepatitis B virus (HBV) infection are not well understood. We screened 36 cytokines and chemokines to determine which were able to reduce the susceptibility of HepaRG cells to HBV infection. Here, we found that pretreatment with IL-1β and TNFα remarkably reduced the host cell susceptibility to HBV infection. This effect was mediated by activation of the NF-κB signaling pathway. A cytidine deaminase, activation-induced cytidine deaminase (AID), was up-regulated by both IL-1β and TNFα in a variety of hepatocyte cell lines and primary human hepatocytes. Another deaminase APOBEC3G was not induced by these proinflammatory cytokines. Knockdown of AID expression impaired the anti-HBV effect of IL-1β, and overexpression of AID antagonized HBV infection, suggesting that AID was one of the responsible factors for the anti-HBV activity of IL-1/TNFα. Although AID induced hypermutation of HBV DNA, this activity was dispensable for the anti-HBV activity. The antiviral effect of IL-1/TNFα was also observed on different HBV genotypes but not on hepatitis C virus. These results demonstrate that proinflammatory cytokines IL-1/TNFα trigger a novel antiviral mechanism involving AID to regulate host cell permissiveness to HBV infection.

## Teaser

**Background:** Cytokines and host factors triggering innate immunity against hepatitis B virus (HBV) are not well understood.

**Results:** IL-1 and TNFα induced cytidine deaminase AID, an anti-HBV host factor, and reduced HBV infection into hepatocytes.

**Conclusion:** IL-1/TNFα reduced host susceptibility to HBV infection through AID up-regulation.

**Significance:** Proinflammatory cytokines modulate HBV infection through a novel innate immune pathway involving AID.

## Introduction

The intracellular immune response can eliminate pathogens from a host, and host cells possess different mechanisms to counteract viral infection depending on the virus type. Human immunodeficiency virus (HIV) infection is restricted by cellular proteins designated as restriction factors, including APOBEC3G (A3G),^3^ TRIM5α, tetherin/BST-2, and SAMHD1 (1, 2). All of these factors can be induced by stimulation with interferon (IFN). Hepatitis C virus (HCV) is eliminated by type I and III IFNs derived from dendritic cells or infected hepatocytes (3–6). In hepatocytes, this process involves a series of antiviral factors that are downstream genes of IFN, IFN-stimulated genes (ISGs). Influenza virus spread and virulence is inhibited by cytokines such as IFNs and TNFα. Responsive genes for these mechanisms include IFN-induced cellular Mx proteins that are dynamin-like GTPases (7, 8). However, these cytokine-induced antiviral immune responses are poorly understood in hepatitis B virus (HBV) infection.

HBV infection is a worldwide health problem affecting more than 350 million people and is a major cause of the development of liver cirrhosis and hepatocellular carcinoma (9–11). During the course of infection, a number of cytokines and chemokines are up-regulated in HBV-infected patients, including IFNα/γ/λ, TNFα, IL-1, IL-6, IL-10, IL-12, IL-15, and IL-8 (12–15). Some of these cytokines are reported to suppress HBV replication (3, 16–21). In particular, type I, II, and III IFNs suppress the replication of HBV *in vitro* and *in vivo* (19, 20, 22–26). Although one of the downstream genes of IFN, *A3G*, has the potential to reduce HBV replication (27–34), it is still under discussion whether this protein is responsible for the anti-HBV activity of type I IFN, because it has been previously reported by Trono and co-workers (28, 35) that the induction of A3G does not explain the IFN-induced inhibition of HBV replication. Moreover, these studies were carried out using an HBV transgene that only reproduces a portion of the whole HBV life cycle, mainly focusing on intracellular HBV replication.

Here, we screened for cytokines and chemokines that affected HBV infection in HepaRG cells, a human hepatocyte cell line susceptible to HBV infection and reproducing the whole HBV life cycle (36, 37). IL-1 and TNFα decreased the host cell permissiveness to HBV infection, and this effect was at least partly mediated by the induction of activation-induced cytidine deaminase (AID). The anti-HBV activity of IL-1/TNFα was mechanistically different from that of IFNα. This study presents the activity of IL-1/TNFα to suppress HBV infection into hepatocytes independent of the effect on immune cells and the physiological role of AID in this machinery. Moreover, as far as we know, this is the first report to show the AID function to inhibit the infection of human pathogenic virus.

## EXPERIMENTAL PROCEDURES

### 

#### 

##### Reagents

All cytokines were purchased from PeproTech or R & D Systems. Heparin was obtained from Mochida Pharmaceutical. Lamivudine, PD98059, SP600125, SB203580, and Bay11-7082 were obtained from Sigma. Entecavir was obtained from Santa Cruz Biotechnology. BMS-345541 and 6-amino-4-(4-phenoxyphenylethylamino)quinazoline (QNZ) were purchased from Merck.

##### Cell Culture

HepaRG cells (Biopredic) were cultured with Williams' medium E (Invitrogen) supplemented with 2 mm l-glutamine, 200 units/ml penicillin, 200 μg/ml streptomycin, 10% FBS, 5 μg/ml insulin (Wako), 20 ng/ml EGF (PeproTech), 50 μm hydrocortisone (Sigma), and 2% DMSO (Sigma). HepG2, HepAD38 (kindly provided by Dr. Seeger at Fox Chase Cancer Center) (38), and HepG2.2.15 cells (a kind gift from Dr. Urban at Heidelberg University) (39) were cultured with DMEM/F-12 + GlutaMAX (Invitrogen) supplemented with 10 mm HEPES (Invitrogen), 200 units/ml penicillin, 200 μg/ml streptomycin, 10% FBS, 50 μm hydrocortisone, and 5 μg/ml insulin in the presence (HepAD38 and HepG2.2.15) or absence (HepG2) of 400 μg/ml G418 (Nacalai Tesque). HepAD38 cells were cultured with 0.3 μg/ml tetracycline when terminating HBV induction. Huh-7.5.1 cells (kindly provided from Dr. Chisari at Scripps Research Institute) were cultured as described previously (40). Primary human hepatocytes (PHH) isolated from urokinase-type plasminogen activator transgenic/SCID mice inoculated with PHH (PhoenixBio) or purchased from Lonza were cultured with DMEM supplemented with 20 mm HEPES, 100 units/ml penicillin, 100 μg/ml streptomycin, 10% FBS, and 44 mm NaHCO_3_ or with 1 mm pyruvate, nonessential amino acids, 20 mm HEPES, 200 units/ml penicillin, 200 μg/ml streptomycin, 10% FBS, 0.25 μg/ml insulin (Wako), 5 ng/ml EGF, and 50 nm dexamethasone.

##### HBV Preparation and Infection

HBV used in this study was mainly derived from HepAD38 cells, which is classified as genotype D (38). Media from HepAD38 cells at days 7–31 post-induction of HBV by depletion of tetracycline were recovered every 3 days. Media were cleared through a 0.45-μm filter and precipitated with 10% PEG8000 and 2.3% NaCl. The precipitates were washed and resuspended with medium at ∼200-fold concentration. The HBV DNA was quantified by real time PCR. HBV genotype A and C in Fig. 7*B* was recovered from the media of HepG2 cells transfected with the plasmid pHBV/Aeus and pHBV/C-AT (41).

HepaRG cells were infected with HBV at 2000 (Fig. 7*B*) or 6000 (other figures) genome equivalent (GEq)/cell in the presence of 4% PEG8000 for 16 h as described previously (36). Urban and co-workers (42) reported that more than 10^3^ GEq/cell amount of HBV derived from HepAD38 or HepG2.2.15 cells (*i.e.* 1.25–40 × 10^4^ GEq/cell) as inoculum was required for efficient infection into HepaRG cells. The anti-HBV effect of IL-1/TNFα shown in this study was also observed when inoculated with HBV at 300 GEq/cell (data not shown).

##### Extraction of DNA and RNA

HBV DNA was extracted from the cells or from the medium using a DNA kit (Qiagen) according to the manufacturer's protocol. Total RNA was recovered with RNeasy mini kit (Qiagen) according to the manufacturer's protocol.

##### Real Time PCR and RT-PCR

HBV DNA was quantified by real time PCR analysis using the primer set 5′-ACTCACCAACCTCCTGTCCT-3′ and 5′-GACAAACGGGCAACATACCT-3′ and probe 5′-carboxyfluorescein (FAM)-TATCGCTGGATGTGTCTGCGGCGT-carboxytetramethylrhodamine (TAMRA)-3′ (43). The PCR was performed at 50 °C for 2 min, 94 °C for 10 min, and 50 cycles of 94 °C for 15 s and 60 °C for 1 min. Detection of cccDNA was achieved using 5′-CGTCTGTGCCTTCTCATCTGC-3′ and 5′-GCACAGCTTGGAGGCTTGAA-3′ as primers and 5′-CTGTAGGCATAAATTGGT (MGB)-3′ as a probe (44). This primer-probe set theoretically detected neither relaxed circular DNA nor HBV DNA integrated into host genome but can capture cccDNA as described previously (44). For quantification of cellular mRNA, cDNA was synthesized from extracted RNA using SuperScriptIII (Invitrogen), followed by PCR with TaqMan Gene Expression Master Mix (Applied Biosystems) and primer-probe set (TaqMan Gene Expression Assay, Applied Biosystems) or with Power SYBR Green PCR Master Mix (Applied Biosystems) and 5′-AAATGTCCGCTGGGCTAAGG-3′ and 5′-GGAGGAAGAGCAATTCCACGT-3′ as primers for *AID*.

RT-PCR was performed as described previously (45) using a one-step RNA PCR kit (Takara). Primers for amplifying each gene were as follows: 5′-CTCTGAGGTTTAGCATTTCA-3′ and 5′-CTCCAGGTCCAAAATGAATA-3′ for *cIAP*; 5′-GCAGATTTATCAACGGCTTT-3′ and 5′-CAGTTTTCCACCACAACAAA-3′ for *XIAP*; 5′-TAGCCAACATGTCCTCACAGAC-3′ and 5′-TCTTCTACCACTGGTTTCATGC-3′ for *ISG56*; 5′-GCCTTTTCATCCAAATGGAATTC-3′ and 5′-GAAATCTGTTCTGGGCTCATG-3′ for *PKR*; and 5′-CCATGGAGAAGGCTGGGG-3′ and 5′-CAAAGTTGTCATGGATGACC-3′ for *GAPDH*, respectively.

##### ELISA

HBs protein was quantified by ELISA using plates incubated at 4 °C overnight with a sheep anti-HBs antibody at 1:5000 dilution (Maxisorp nunc-immuno plate, Nunc catalog no. 439454) followed by coating with 0.2% BSA, 0.02% NaN_3_, 1× PBS at 4 °C until use. Samples were incubated with the plates for 2 h and after washing with TBST four times, horseradish peroxidase-labeled rabbit anti-HBs antibody was added for 2 h. The substrate solution (HCV core ELISA kit: Ortho) was reacted for 30 min before the *A*_450_ values were measured.

##### Indirect Immunofluorescence Analysis

Indirect immunofluorescence analysis was performed essentially as described previously (45). After fixation with 4% paraformaldehyde and permeabilization with 0.3% Triton X-100, an anti-HBc antibody (DAKO, catalog no. B0586) was used as the primary antibody.

##### MTT Assay

The MTT assay was performed as described previously (46).

##### Immunoblot Analysis

Immunoblot analysis was performed as described previously (47). The polyclonal antibody against AID was generated using a peptide derived from AID protein as an immunogen as described previously for preparation of the anti-AID antibody 1 (48). The specificity of the antibody was described previously (48, 49).

##### Lentiviral Vector-mediated Gene Transduction

Lentivirus carrying shRNAs was prepared with 293T cells transfected with expression plasmids for HIV-1 Gag-Pol, VSV G, and shRNAs (sh-control, sh-cyclophilin A, sh-AID(1), sh-AID(2); Mission shRNA) (Sigma) with Lipofectamine 2000 (Invitrogen). Recovered lentiviral vector was transduced into HepaRG cells followed by selection with 1.5 μg/ml puromycin. Lentivirus overexpressing AID, AID mutant, A3G, or the control lentivirus was recovered using expression plasmids for HIV-1 Gag-Pol, Rev, VSV G, and the corresponding expression vector as described previously (50).

##### Southern Blot Analysis

Southern blot was performed as described previously (41). After digestion of free nucleic acids with DNase I and RNase A, cell lysates were digested with proteinase K, and HBV DNA in the core particles was extracted with phenol/chloroform, followed by isopropyl alcohol precipitation. Probe was prepared by cutting pHBV/D-IND60 (41) with SacII and BspHI to generate a full-length HBV DNA probe and labeled with AlkPhos direct labeling reagents (GE Healthcare). Labeled bands were visualized with CDP-star detection reagent (GE Healthcare).

##### Quantification of Nucleocapsid-associated HBV RNA

After digestion of free nucleic acids with DNase I and RNase A, nucleocapsid was precipitated with PEG8000 (41). Total RNA was then extracted from the resuspended precipitates. HBV RNA was quantified by real time RT-PCR with 5′-TCCCTCGCCTCGCAGACG-3′ and 5′-GTTTCCCACCTTATGAGTC-3′ as primers with Power SYBR Green PCR Master Mix (Applied Biosystems).

##### Co-immunoprecipitation Assay

Co-immunoprecipitation assay was essentially performed as described (45).

##### Differential DNA Denaturation PCR

Differential DNA denaturation PCR was performed as described previously (51).

##### Reporter Assay

**FIGURE 1.**
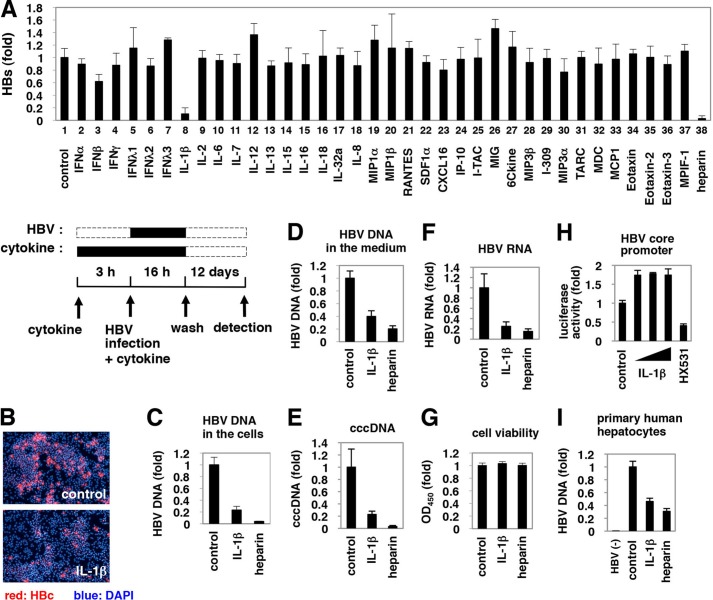
**Suppression of HBV infection by IL-1β.** *A, upper graph*, HepaRG cells were pretreated with cytokines at 100 ng/ml (except for IFNα and IFNβ at 100 IU/ml) or heparin at 25 units/ml as a positive control or were left untreated (*control*) for 3 h and then infected with HBV in the presence of each stimuli for 16 h. After washing, cells were cultured in normal growth medium for 12 days. HBs protein secreted into the medium was quantified by ELISA. *Lower scheme* indicates the treatment procedure for HepaRG cells. *Black* and *dashed line boxes* indicate the periods with and without treatment, respectively. *B–G* and *I,* HepaRG cells (*B–G*) or PHH (*I*) were treated as shown in *A* with or without 100 ng/ml IL-1β or 25 units/ml heparin as a positive control. HBc protein in the cells (*red*) was detected by indirect immunofluorescence analysis, and the nucleus was stained with DAPI (*blue*) at 12 days post-infection (*B*). HBV DNA (*C* and *I*), cccDNA (*E*), and HBV RNA (*F*) in the cells as well as HBV DNA in the medium (*D*) were detected. Cell viability was quantified by MTT assay (*G*). *HBV*(−) in *I* indicates uninfected cells. All of the data, except in *I*, are based on the average of three independent experiments. *I* shows the average results from one representative experiment, but the reproducibility of the data were confirmed in three independent experiments. *H,* reporter plasmid carrying the HBV core promoter was transfected with HepG2 cells and then treated with or without IL-1β (1, 10, and 100 ng/ml) and an retinoid X receptor antagonist HX531 as a positive control for 6 h. Luciferase activity was measured.

DNA transfection was performed with pNF-κB-luc or pISRE-TA-luc (Stratagene) and pRL-TK (Promega), which express firefly luciferase driven by NF-κB or ISRE and *Renilla* luciferase by herpes simplex virus thymidine kinase promoter, respectively, and Polyethylenimine Max (Polysciences Inc., catalog no. 24765). After compound or cytokine treatment, cells were lysed, and luciferase activities were measured as described previously (52). A reporter carrying HBV core promoter was constructed by inserting the DNA fragment (1413–1788 nucleotide number) of HBV DNA (D-IND60) into pGL4.28 vector (Promega) (41). In the reporter assay using this construct (Fig. 1*H*), HX531, a retinoid X receptor antagonist was used as a positive control as retinoid X receptor was involved in the transcription from the core promoter (53).

## RESULTS

### 

#### 

##### IL-1 Reduced Host Cell Susceptibility to HBV Infection

To evaluate the effect of cytokines and chemokines on susceptibility to HBV infection, we treated HepaRG cells (36) with cytokines for 3 h prior to and 16 h during HBV infection, followed by culture without stimuli for an additional 12 days (Fig. 1*A*, *lower scheme*). Heparin, a competitive inhibitor of HBV attachment (54), was used as a positive control and decreased secretion of the viral envelope surface protein (HBs) from HBV-infected cells (Fig. 1*A, upper graph, lane 38*), which suggests a successful HBV infection in this experiment. Examination of 36 cytokines and chemokines revealed that IL-1β drastically decreased protein secretion from HBs (Fig. 1*A, upper graph, lane 8*). Although IFNs had a strong anti-HBV effect by a continuous treatment after HBV infection (Fig. 3*C, panel b,* and data not shown), they had only a limited effect in this screening where cytokines were only pretreated and cotreated with HBV (Fig. 1*A*, *lanes 2–7*). HBc protein expression (Fig. 1*B*) and HBV DNA (Fig. 1*C*) in the cells and medium (Fig. 1*D*) were significantly decreased by treatment with IL-1β without cytotoxicity (Fig. 1*G*). HBV cccDNA and HBV RNA was also decreased in infected cells treated with IL-1β (Fig. 1, *E* and *F*). IL-1β did not decrease HBV core promoter activity at least in HepG2 cells (Fig. 1*H*). These results suggest that IL-1β suppressed HBV infection to HepaRG cells. IL-1β did not decrease the expression of sodium taurocholate cotransporting polypeptide (*NTCP*), a recently reported HBV entry receptor (data not shown) (55). Similar results were obtained using primary human hepatocytes (Fig. 1*I*).

##### NF-κB Signaling Was Critical for Anti-HBV Activity

**FIGURE 2.**
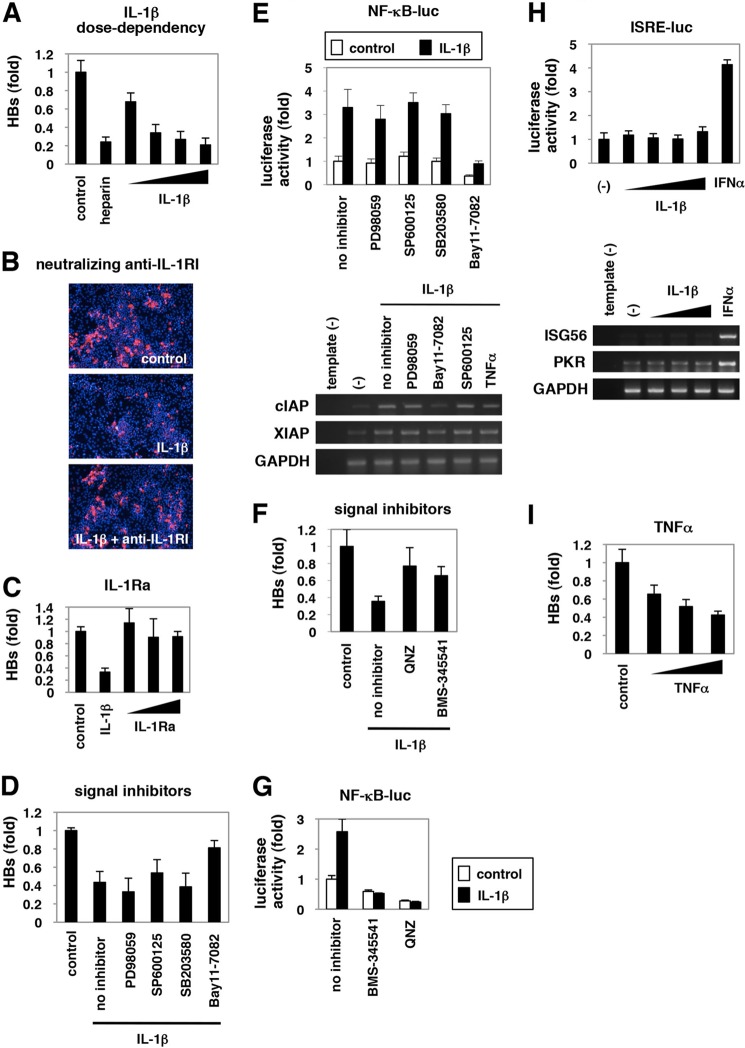
**NF-κB activation triggered by IL-1 and TNFα was critical for anti-HBV activity.** *A–D, F,* and *I,* HepaRG cells were left untreated (*control*) or treated with varying concentrations of IL-1β (1, 10, 30, and 100 ng/ml) or 25 units/ml heparin (*A*), with 30 ng/ml IL-1β together with or without a neutralizing anti-IL-1RI antibody at 20 μg/ml (*B*), with 10 ng/ml IL-1β or varying concentrations of IL-1Ra (10, 30, and 100 ng/ml) (*C*), with 3 ng/ml IL-1β together with or without PD98059, SP600125, SB203580, or Bay11-7082 (*D*), or QNZ or BMS-345541 (*F*), or with TNFα (10, 100, and 300 ng/ml) (*I*) according to the treatment schedule shown in Fig. 1*A*. HBV infection was monitored by HBs protein secretion into the medium in *A*, *C, D, F,* and *I* and with HBc protein in the cells in *B. E, G,* and *H,* NF-κB (*E* and *G*) and ISRE activity (*H*) were measured by reporter assay in the cells transfected with the reporter plasmid expressing luciferase driven from five tandem repeats of NF-κB elements (*E, upper graph,* and *G*) or ISRE (*H, upper graph*) or by RT-PCR in the cells (*E* and *H, lower panels*) upon signaling inhibitors used in *D* and *F* together with or without IL-1β (*E* and *G*), or upon IL-1β (10, 30, and 100 ng/ml) or IFNα 100 IU/ml as a positive control (*H*) for 6 h. The *white* and *black bars* in the *upper graph* of *E* and *G* show the data in the absence or presence of IL-1β, respectively. Bands for mRNA for *cIAP*, *XIAP*, and *GAPDH* (*E*) or *ISG56*, *PKR*, or *GAPDH* (*H*) are presented in the *lower panels*. All of the data are based on averages of three independent experiments.

As shown in Fig. 2*A*, IL-1β suppressed HBV infection in a dose-dependent manner. This anti-HBV effect was reversed by cotreatment with a neutralizing antibody for the IL-1 receptor, IL-1RI (Fig. 2*B*), suggesting that receptor engagement was required for anti-HBV activity. IL-1Ra is a natural antagonist that associates with IL-1RI but does not trigger downstream signal transduction (56). Treatment with IL-1Ra did not decrease HBV infectivity (Fig. 2*C*), suggesting that signal transduction triggered by IL-1 was required for anti-HBV activity.

To identify the signal transduction pathway essential for anti-HBV activity, we treated HepaRG cells with PD98059, SP600125, SB203580, and Bay11-7082, which are inhibitors for MEK, JNK, p38, and NF-κB, respectively (57). As shown in Fig. 2*D*, only cotreatment with Bay11-7082 significantly removed the anti-HBV effect of IL-1β. Luciferase assay and RT-PCR analysis indicated that Bay11-7082, but not other inhibitors, blocked the transactivation of NF-κB (Fig. 2*E*, *upper panels*) and NF-κB downstream genes, *cIAP* and *XIAP* (Fig. 2*E*, *lower panels*). Additional NF-kB inhibitors, BMS-345541 and QNZ (Fig. 2*G*), also reversed the anti-HBV effect of IL-1β (Fig. 2*F*). These data suggest a critical role for NF-κB activation in the anti-HBV activity. Additionally, IL-1β did not augment the activity of interferon sensitivity-responsive element (ISRE) and mRNAs for ISGs, *ISG56*, and double-stranded RNA-dependent protein kinase (*PKR*) in HepaRG cells (Fig. 2*H*), suggesting that the anti-HBV activity is independent of ISG up-regulation. TNFα, another cytokine that activates NF-κB signaling (Fig. 2*E*, *lower panels*), also inhibited HBV infection (Fig. 2*I*). Thus, NF-κB activation in host hepatocytes was critical for the anti-HBV activity of proinflammatory cytokines.

##### Early Phase of HBV Infection as Well as HBV Replication Were Impaired by IL-1 Treatment

**FIGURE 3.**
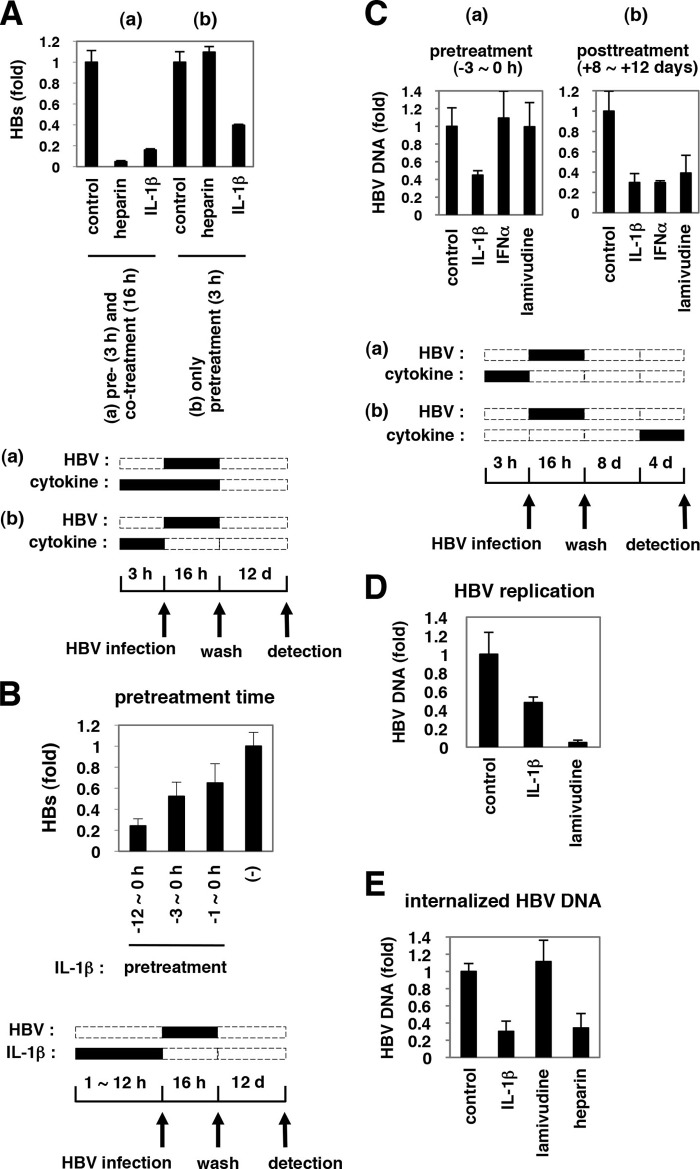
**Defining the steps of the HBV life cycle targeted by IL-1β.** *A,* HepaRG cells were pretreated with IL-1β or heparin for 3 h and then infected with HBV in the presence (*A, panel a*) or absence (*A, panel b*) of IL-1β or heparin for 16 h. HBV infection was monitored with HBs protein secretion from the infected cells. Only pretreatment with IL-1β and not heparin could inhibit HBV infectivity. *d*, day. *B,* HepaRG cells were pretreated with IL-1β or left untreated (−) for the indicated time (*h*) and infected with HBV without IL-1β. Anti-HBV activity was amplified by a prolonged treatment time. *C, panel a,* HepaRG cells were pretreated with 10 ng/ml IL-1β, 100 IU/ml IFNα, or 1 μm lamivudine for 3 h, followed by infection with HBV for 16 h in the absence of cytokines (*pretreatment*). *C, panel b,* HepaRG cells were infected with HBV for 16 h without pretreatment. After washing out the input virus, cells were cultured in normal medium for the first 8 days and then cultured with IL-1β, IFNα, or lamivudine for the following 4 days (*post-treatment*). HBV DNA in the cells was measured by real time PCR. IL-1β showed an anti-HBV activity in both pretreatment and post-treatment, although an anti-HBV effect of IFNα was seen only with post-treatment. *D,* HepAD38 cells were treated with 100 ng/ml IL-1β or 1 μm lamivudine, or left untreated for 6 days in the absence of tetracycline. HBV replication was evaluated by measurement of HBV DNA in the medium. *E,* HepaRG cells were pretreated with IL-1β, lamivudine, or heparin for 3 h or left untreated and infected with HBV for 16 h in the presence or absence of each compound. After trypsinization and extensive washing of the cells, cellular DNA was immediately recovered to detect HBV DNA. HBV DNA at 16 h post-infection was decreased by treatment with IL-1β but not lamivudine.

Although heparin, an attachment inhibitor, could block HBV infection only if added together with the HBV inoculum, pretreatment with IL-1β before HBV infection was sufficient to show anti-HBV activity (Fig. 3*A, panel b*). This activity was amplified by a prolonged treatment time of up to 12 h (Fig. 3*B*). Intriguingly, HBV cellular DNA was also reduced by IL-1β treatment following HBV infection (Fig. 3*C, panel b*). In contrast, IFNα was not effective by pretreatment (Figs. 3*C, panel a,* and 1*A*), although it did decrease HBV DNA by treatment after HBV infection (Fig. 3*C, panel b*), consistent with previous reports that IFNα can suppress HBV replication (19, 20, 26). Thus, the anti-HBV activity of IL-1β is likely to be mechanistically different from that of IFNα.

The HBV life cycle can be divided into at least two phases as follows: 1) the early phase of infection that includes attachment, entry, nuclear import, and cccDNA formation; and 2) the late phase representing HBV replication, including transcription, assembly, reverse transcription, DNA synthesis, and viral release (58). The early phase of HBV infection is not supported, but HBV DNAs persistently replicate in HepAD38 cells in the presence of tetracycline (38). IL-1β decreased the HBV DNA levels in HepAD38 cells (Fig. 3*D*), suggesting suppression of HBV replication. In addition, to examine the early phase preceding HBV replication, we infected HepaRG cells with HBV in the presence of IL-1β for 16 h and then immediately recovered cellular DNA in the trypsinized cells for quantification of HBV DNA (Fig. 3*E*). This procedure likely detected HBV DNA that had been internalized and evaded the host restriction before initiation of HBV replication because lamivudine showed no effect on the amount of DNA detected (Fig. 3*E*). In this experiment, IL-1β significantly decreased HBV DNA (Fig. 3*E*). cccDNA was also decreased by IL-1β, suggesting that the early phase of HBV infection before cccDNA formation was also interrupted by IL-1β.

##### IL-1 and TNFα Induced the Expression of AID

**FIGURE 4.**
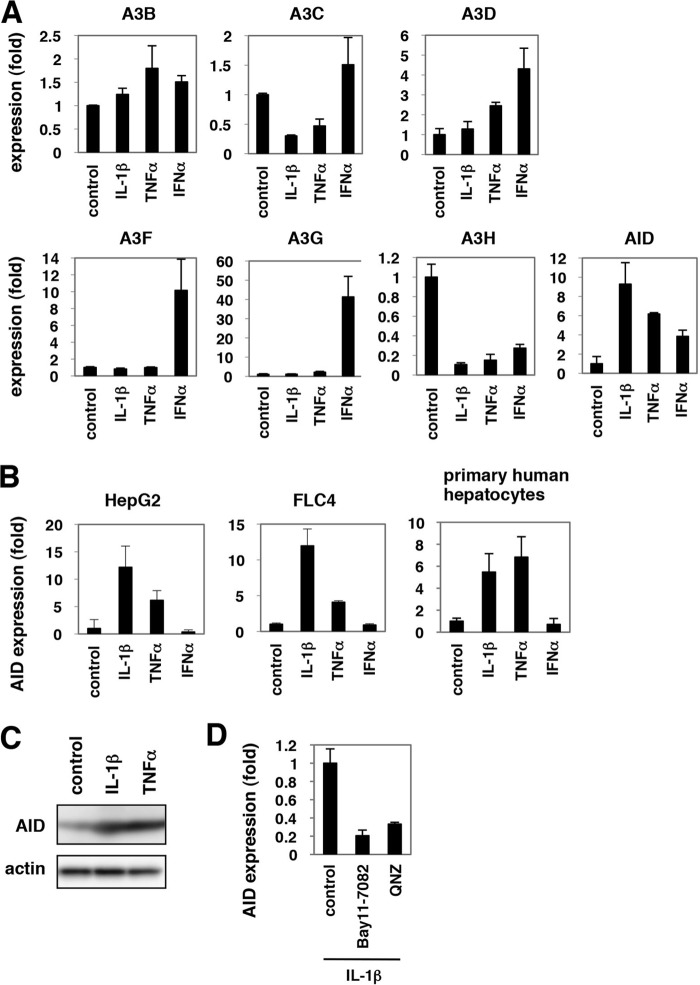
**AID expression was induced by IL-1β and TNFα.** *A,* mRNAs for *A3B, -C, -D, -F, -G, -H* and *AID* were quantified by real time RT-PCR analysis in HepaRG cells treated with 100 ng/ml IL-1β, 100 ng/ml TNFα, or 100 IU/ml IFNα for 12 h or left untreated. *Graphs* show the relative expression levels compared with the controls set at 1. *B, AID* mRNA was detected in HepG2, FLC4 cells, and PHH treated with IL-1β, TNFα, or IFNα or left untreated. Induction of AID by IL-1β and TNFα was observed in HepG2 and FLC4 cells and primary human hepatocytes. *C,* AID protein (*upper panel*) and actin levels as an internal control (*lower panel*) were examined by immunoblot of primary human hepatocytes treated with IL-1β or TNFα or left untreated. *D,* AID mRNA was detected in PHH treated with 100 ng/ml IL-1β in the presence or absence of NF-κB inhibitors, Bay11-7082, or QNZ for 12 h.

The innate immune pathway against HBV infection remains largely unknown. Recently, accumulating evidence suggested that several APOBEC family proteins, especially A3G, suppressed HBV replication when overexpressed (27–33). In contrast, there was no report available suggesting the anti-HBV function of other restriction factors against HIV, TRIM5α, tetherin/BST-2, and SAMHD1. We then investigated APOBEC family proteins as a candidate for an anti-HBV effector. The APOBEC family includes APOBEC1 (A1), A2, A3s, A4, and AID (59). Because some of these proteins are reported to be up-regulated in cytokine-stimulated hepatocytes (27, 28, 60, 61), we examined the expression of these genes in cells treated with IL-1β, TNFα, and IFNα as a control for 12 h. The mRNA levels of *A1*, *A2*, and *A3A* were below the detection threshold. *A3G* and *A3F* mRNA were significantly expressed in HepaRG cells, and their expression levels were remarkably increased by IFNα treatment (Fig. 4*A*), as observed in other reports (27, 28, 61). IL-1β and TNFα did not significantly up-regulate A3s, and only AID was up-regulated 6–10-fold by both cytokines (Fig. 4*A*). Induction of A3s by both IL-1β and TNFα was not observed at any time point examined until 12 h (data not shown). In contrast, induction of *AID* mRNA by IL-1β and TNFα was conserved in human hepatocyte cell lines, such as HepG2 and FLC4 cells, and in primary human hepatocytes (Fig. 4*B*). AID protein production was also increased in primary human hepatocytes by treatment with IL-1β and TNFα (Fig. 4*C*). This AID induction by IL-1β was suggested to be NF-κB-dependent, because the up-regulation of *AID* mRNA was canceled by addition of NF-κB inhibitors, Bay11-7082 or QNZ (Fig. 4*D*).

##### AID Played a Significant Role in the IL-1-mediated restriction of HBV

**FIGURE 5.**
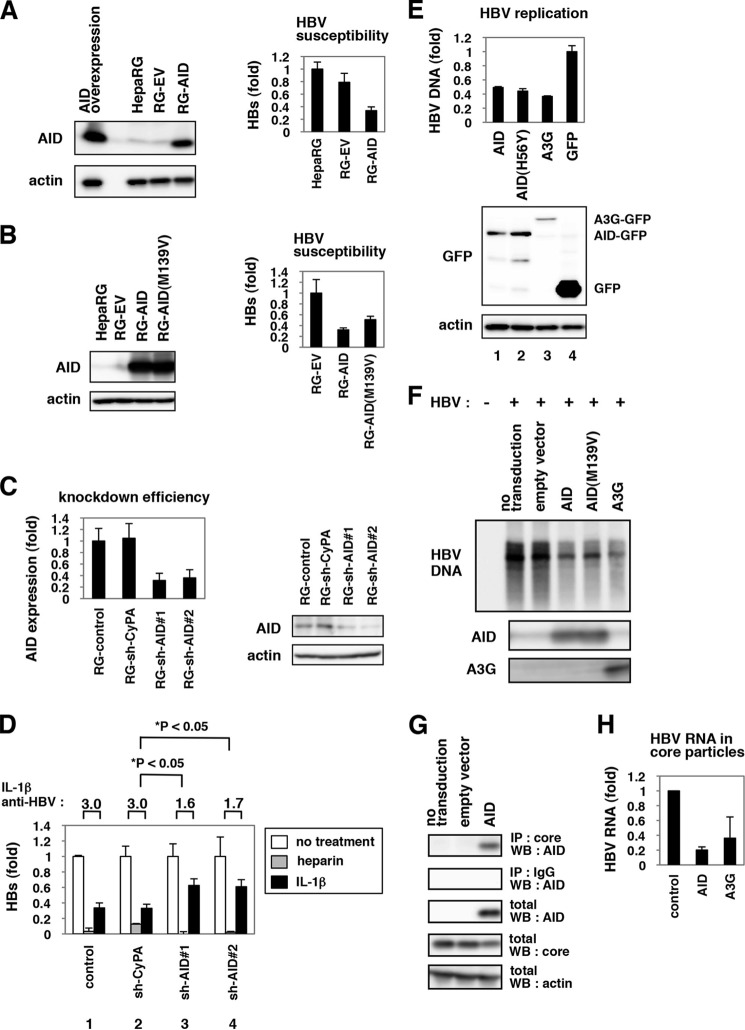
**AID played a significant role in IL-1-mediated anti-HBV activity.** *A* and *B, left panels,* HepaRG cells were transduced with a lentiviral vector carrying the expression plasmid for AID (*RG-AID*), AID(M139V) mutant (*RG-AID*(*M139V*)) (*B*), or the control vector (*RG-EV*). Protein expression for AID (*upper panel*) and actin (*lower panel*) in these cells, the parental HepaRG cells (*HepaRG*), and those transiently transfected with AID expression plasmid (*AID overexpression*) (*A*) was examined by immunoblot. *Right panels,* these cells were infected with HBV followed by detection of secreted HBs protein as Fig. 1*A*. AID-transduced cells were less susceptible to HBV infection. *C,* HepaRG cells were transduced with lentiviral vector carrying shRNAs for AID (*RG-shAID#1* and *RG-shAID#2*) or for cyclophilin A (*RG-shCyPA*) as a control. AID mRNA (*left panel*) and protein (*right panel*) were quantified by real time RT-PCR and immunoblot analysis. *D,* cells produced in *C* were infected with HBV in the absence or presence of IL-1β or heparin, and HBs was detected in the medium as in Fig. 1*A* to examine the anti-HBV effect of IL-1β and heparin. The fold reduction of HBV infection by IL-1β treatment is shown as *IL-1*β *anti-HBV* above the *graph*. The *white*, *gray*, and *black bars* indicate HBs value of the cells without treatment and with heparin and IL-1β treatment, respectively. The anti-HBV activity of IL-1β but not heparin was reduced in the AID-knockdown cells. *E,* AID and its mutant suppressed HBV replication. HepG2 cells were cotransfected with GFP-tagged AID, AID(H56Y), A3G, and GFP itself along with an HBV-encoding plasmid. Following 3 days, cytoplasmic nucleocapsid HBV DNA was quantified (*upper graph*), and the overexpressed proteins as well as actin were detected (*lower panels*). *F,* lentiviral vectors carrying AID, AID(M139V) mutant, A3G, or an empty vector (*empty vector*) were transduced or left untransduced (*no transduction*) into HepG2.2.15 cells. Nucleocapsid associated HBV DNA in these cells or in HepG2 cells (*HBV*−) was detected by Southern blot (*upper panel*). AID (*middle panel*) and A3G protein (*lower panel*) were also detected by immunoblot. *G,* HBV core interacted with AID. HepAD38 cells transduced without (*no transduction*) or with AID-expressing vector or the empty vector (*empty vector*) were lysed and treated with anti-core antibody (*1st panel*) or control normal IgG (*2nd panel*) for immunoprecipitation (*IP*). Total fraction without immunoprecipitation (*3rd* to *5th panels*) was also recovered to detect AID (*1st* to *3rd panels*), HBV core (*5th panel*), and actin (*5th panel*) by immunoblot. *WB,* Western blot. *H,* HBV RNA in core particles was extracted as shown under “Experimental Procedures” in HepG2 cells overexpressing HBV DNA together with or without AID or A3G.

To examine the function of AID during HBV infection, we transduced AID ectopically into HepaRG cells using a lentiviral vector (Fig. 5*A*, *left panel*). The susceptibility of these AID-overexpressing cells to HBV was decreased by approximately one-third compared with the parental or empty vector-transduced HepaRG cells (Fig. 5*A*, *right panel*), suggesting that AID can restrict HBV infection. An AID mutant AID(M139V), with reported diminished activity to support class switching (48), also decreased the susceptibility to HBV infection, although the reduction in HBV susceptibility was moderate compared with the case of the wild type AID (Fig. 5*B*).

To examine the relevance of endogenous AID in the anti-HBV activity of IL-1, we transduced a lentiviral vector carrying a short hairpin RNA (shRNA) against AID (sh-AID) or a nonrelevant protein cyclophilin A (Fig. 5*C*), and we observed the anti-HBV activity of IL-1β in these cells. IL-1β decreased HBV infection in the control and sh-cyclophilin A -transduced cells by ∼3.0-fold as determined by HBs secretion (Fig. 5*D*, *lanes 1* and *2, black bars*). In contrast, anti-HBV activity of IL-1β was limited to only 1.6–1.7-fold in the cells transduced with sh-AIDs (Fig. 5*D*, *lanes 3* and *4, black bars*). Such relieved anti-HBV activity following AID knockdown was not observed in the case for heparin treatment (Fig. 5*D*, *lanes 1–4, gray bars*). Similar results were obtained by monitoring intracellular HBV DNA after infection (data not shown). Although the anti-HBV effect of IL-1β was not completely blunted, these data suggest that AID plays a significant role in mediating the anti-HBV effect of IL-1β.

Similar observations were obtained in HBV-replicating cells overexpressing AID (Fig. 5, *E* and *F*). Core particle-associated HBV DNA in HepG2 cells transfected with an HBV-encoding plasmid was decreased by overexpression with AID as well as with A3G (Fig. 5*E*, *lanes 1* and *3*). Intriguingly, HBV DNA in core particles was also decreased by expression of an AID mutant AID(H56Y), which contains a mutation in the cytidine deaminase motif and is derived from a class switch deficiency patient (Fig. 5*E*, *lane 2*) (48). Southern blot also showed that the HBV rcDNA level in HepG2.2.15 cells was reduced by transduction with AID and another mutant AID(M139V), with diminished activity to support class switching (Fig. 5*F*) (48). These data suggest that AID could suppress HBV replication, and this restriction activity can be still observed with reduced enzymatic activity. In addition, AID was shown to interact with HBV core protein by coimmunoprecipitation assay (Fig. 5*G*). Moreover, overexpression of AID reduced the levels for nucleocapsid-associated HBV RNA (Fig. 5*H*). These results further suggest an antiviral activity of AID against HBV replication.

##### AID Could Induce Hypermutation of HBV DNA

**FIGURE 6.**
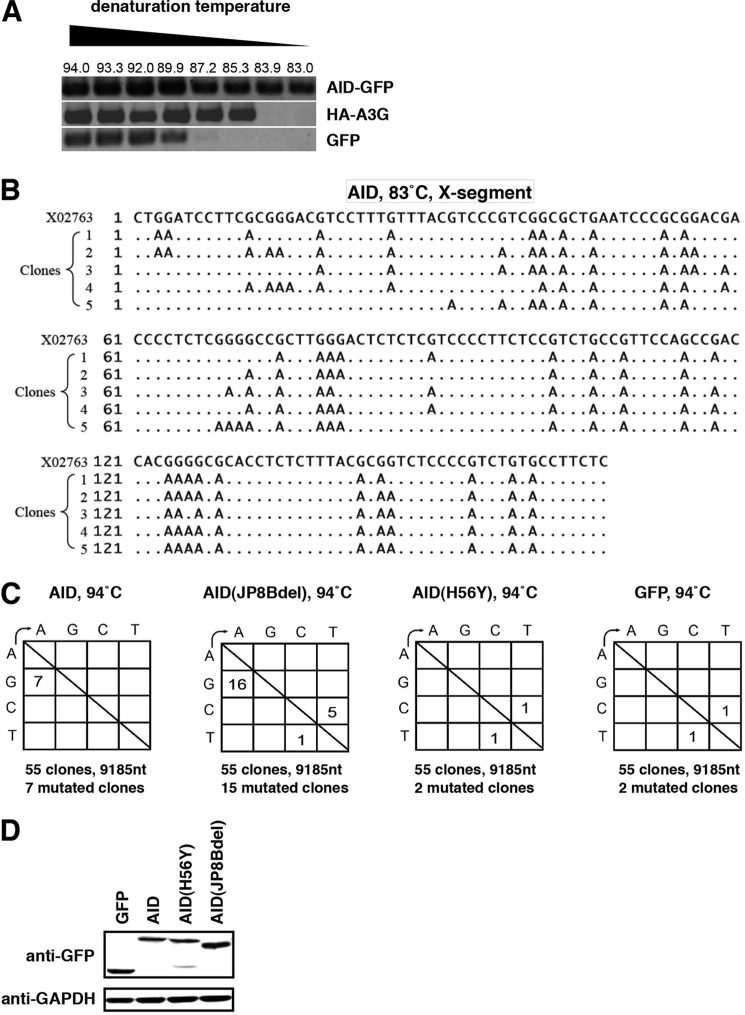
**AID could induce hypermutation of HBV DNA.** *A* and *B,* HepG2 cells were cotransfected with an expression vector for GFP-tagged AID, HA-tagged A3G, or GFP along with an HBV-encoding plasmid. 3 days after transfection, nucleocapsid-associated HBV DNA was extracted, and differential DNA denaturation PCR was performed to amplify the X gene segments. The *numbers above* the panels in *A* show denaturing temperatures. The X gene fragment amplified at 83 °C in the AID sample was cloned in to a T vector and sequenced in *B*. Alignment of independent five clones with reference sequence (X02763) is indicated. *C,* AID and its mutant (JP8Bdel) induced G-to-A and C-to-T hypermutations in HBV DNA. HepG2 cells were transfected with expression vectors of GFP-tagged AID, AID(H56Y), AID(JP8Bdel), or GFP itself together with HBV encoding plasmid. Three days after transfection, cells were harvested, and nucleocapsid-associated HBV DNA was extracted. X gene fragments were amplified at 94 °C and cloned in T vector. 55 clones were sequenced as described under “Experimental Procedures.” The *numbers* indicate the clone numbers carrying the mutation. *D,* expression of GFP, GFP-tagged AID, AID(H56Y), and AID(JP8Bdel) is shown by immunoblot.

Major enzymatic activity for APOBEC family proteins is the introduction of hypermutation in target DNA/RNA, and hypermutation accounts for antiviral activity for A3G against HIV-1 to some extent (2). Several groups reported that APOBEC family proteins could induce hypermutation in HBV DNA (27, 30, 32, 34). Next we asked whether AID could induce hypermutations in HBV DNA. In differential DNA denaturation PCR analysis, a high content of A/T bases introduced by hypermutation decreased denaturation temperatures (51). As shown in Fig. 6*A*, ectopic expression of AID decreased the denaturation temperature of HBV DNA as shown by that of A3G. Sequence analyses of the HBV DNA X region amplified at 83 °C by differential DNA denaturation PCR indicated a massive accumulation of G-to-A mutations by AID (Fig. 6*B*). The frequency of G-to-A mutations was augmented by AID expression (Fig. 6*C*). In this experiment, AID(JP8Bdel), a hyper-active mutant of AID (62), further promoted the accumulation of the G-to-A and C-to-T mutations, although AID(H56Y) showed mutations in HBV DNA equivalent with mock GFP control sample (Fig. 6*C*). Thus, AID had the potential to introduce hypermutation in nucleocapsid-associated HBV DNA.

##### IL-1 Suppressed the Infection of Different HBV Genotypes but Not That of HCV

**FIGURE 7.**
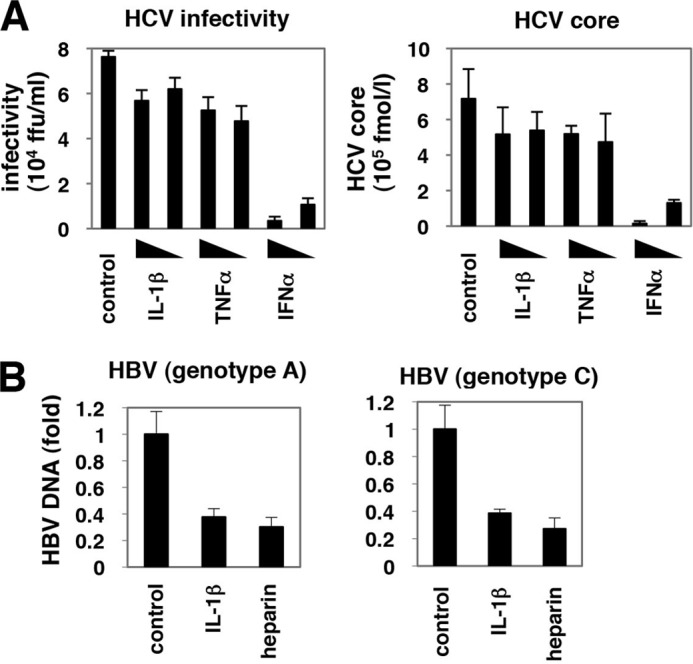
**Antiviral activity of AID was specific to HBV.** *A,* Huh-7.5.1 cells were pretreated with IL-1β, TNFα, or IFNα for 3 h or left untreated and then coincubated with HCV for 4 h. After washing HCV and cytokines and culturing the cells with normal medium for 72 h, the infectivity of HCV (*left panel*) as well as HCV core protein (*right panel*) in the medium was quantified. *B,* HepaRG cells were treated with IL-1β or heparin or left untreated for 3 h prior to and 16 h during infection of HBV genotype A (*left graph*) or C (*right graph*) as shown in Fig. 1*A*. HBV infection was monitored with cellular HBV DNA at 12 days after the infection as Fig. 1*C*.

We examined whether the antiviral activity of IL-1β and TNFα could be generalized to other viruses or was specific to HBV. As shown in Fig. 7*A*, the production of infectious HCV and HCV core proteins in the medium was not significantly altered by treatment with these cytokines in HCV-infected cells, compared to when IFNα was used as a positive control (Fig. 7*A*). In contrast, IL-1 suppressed the infection of HBV genotype A and C into HepaRG cells (Fig. 7*B*) as well as genotype D (Fig. 1*C*). These data suggest that the antiviral activity of proinflammatory cytokines IL-1 and TNFα is specific to HBV.

## DISCUSSION

In this study, cytokine screening revealed that IL-1 and TNFα decreased the host cell susceptibility to HBV infection. This antiviral mechanism is rather unique, given that the intracellular immune response against viruses is typically triggered by IFNs. So far, type I, II, and III IFNs are reported to suppress the replication step of the HBV life cycle (19, 20, 25, 26). In contrast, we suggest that IL-1 and TNFα inhibit the early phase of HBV infection as well as the replication. This is consistent with cumulative clinical evidence suggesting that these proinflammatory cytokines contribute to HBV elimination (63–65). IL-1 and TNFα are generally produced mainly in macrophages and also in other cell types, including T cells and endothelial cells (66). Although the main producer cells of these cytokines in hepatitis B patients are not defined, it has been reported that the secretion of IL-1 and TNFα in nonparenchymal cells were increased by HBV infection into hepatocytes (67). TNFα production in macrophages was augmented by addition of recombinant HBc (68). A number of clinical studies cumulatively show that serum levels of IL-1 and TNFα are increased in hepatitis B patients (12). Recently, it has been a significant clinical problem that HBV reactivates during the course of treatment with immunosuppressants such as anti-TNFα agents (64, 65). Taken together, it is proposed that acute or chronic HBV infection induces IL-1/TNFα from macrophages or other cells in the liver of infected patients, which can directly suppress HBV infection in hepatocytes, in addition to their immunomodulatory effects to the host immune cells. Although IL-1 level in HBV-infected patients varies between papers, Daniels *et al.* (63) reported that the peak IL-1β level in HBV-infected patients was 9–36 ng/ml under Toll-like receptor stimulation, at which concentration IL-1β showed significant anti-HBV effects in this study. In general, downstream genes of NF-κB include a number of antiviral factors such as *viperin*, *iNOS*, and *RANTES* (69). Although some of these genes may function cooperatively for IL-1- and TNFα-induced anti-HBV machinery, our data suggest that AID, at least in part, plays a role in the elimination of HBV that was potentiated by proinflammatory cytokines IL-1 and TNFα.

AID belongs to APOBEC family proteins that share enzyme activity to convert cytidine to uracil in mainly DNA, and occasionally RNA (51, 70, 71). Although AID was initially identified in B cells, chronic inflammation can trigger its expression in hepatocytes (60). The induction of AID was reportedly mediated by NF-κB (60), consistent with the results in this study. Although AID in B cells is essential for class switch recombination and somatic hypermutation of immunoglobulin genes (70, 72), the physiological role of AID in hepatocytes is unknown. Although expression of AID in hepatocytes is still lower than in B cells, AID is reportedly expressed in the liver both in cell culture and *in vivo* settings (34, 60). Our results raise the idea that AID plays a role in innate antiviral immunity. AID also has a role in virus-induced pathogenesis as it was reported to counteract oncogenesis induced by Abelson-murine leukemia virus (73). In addition, AID was reported to restrict L1 retrotransposition, which can predict the role of AID in innate immunity (74). This study is significant in that it revealed a biological function of AID in viral infection itself, linking it to the restriction of a pathogenic human virus. It will be interesting to analyze the role of AID in the infection process of other viruses in the future.

Although the mechanism for AID suppression of the HBV life cycle is the subject of future study, AID possibly targets the early phase of HBV infection, including entry as well as the replication stage, including assembly and reverse transcription (Fig. 3). It has been recently reported that chicken AID reduced cccDNA of duck HBV possibly through targeting cccDNA as well as nucleocapsid-associated HBV DNA (75). This study is likely to support the idea that AID may target cccDNA formed after HBV entry into hepatocytes, and also associates with nucleocapsid-associated HBV DNA during HBV replication, although it is not clear whether the innate immune machinery against HBV/duck HBV is conserved in human and chicken cells. A3G blocked HBV replication through the inhibition of reverse transcriptase (29), packaging of pregenomic RNA (33), and the destabilization of packaged pregenomic RNA (31) independently of its deaminase activity, and it also induced hypermutation of HBV DNA (27, 30, 32, 34). It was recently reported that AID was packaged into the HBV nucleocapsid (51). Moreover, AID induced C-to-T and G-to-A hypermutations in HBV DNA/RNA, although the anti-HBV activity has not been demonstrated so far (51). The hypermutation activity of AID was likely to be dispensable for its anti-HBV replication function (Figs. 5 and 6), as reported for APOBEC3G by several groups (29, 30, 33). Further analysis is required to elucidate the precise mechanisms for AID-mediated suppression of the HBV life cycle.

In conclusion, we have identified that host cell susceptibility to HBV infection is modulated by IL-1 and TNFα, and AID is involved in this machinery. This sheds new light on the link between proinflammatory cytokines and the development of the innate antiviral defense.

## Footnotes

3 The abbreviations used are:

A3G: APOBEC3G
AID: activation-induced cytidine deaminase
HBV: hepatitis B virus
HCV: hepatitis C virus
ISG: IFN-stimulated gene
QNZ: 6-amino-4-(4-phenoxyphenylethylamino)quinazoline
GEq: genome equivalent
PHH: primary human hepatocyte
MTT: 3-(4,5-dimethylthiazol-2-yl)-2,5-diphenyltetrazolium bromide
ISRE: interferon sensitivity-responsive element
cccDNA: covalently closed circular DNA.
